# Profiling Nematode Communities in Unmanaged Flowerbed and Agricultural Field Soils in Japan by DNA Barcode Sequencing

**DOI:** 10.1371/journal.pone.0051785

**Published:** 2012-12-20

**Authors:** Hisashi Morise, Erika Miyazaki, Shoko Yoshimitsu, Toshihiko Eki

**Affiliations:** Molecular Genetics Laboratory, Division of Bioscience and Biotechnology, Department of Environmental and Life Sciences, Toyohashi University of Technology, Tempaku-cho, Toyohashi, Aichi, Japan; Catalan Institute for Water Research (ICRA), Spain

## Abstract

Soil nematodes play crucial roles in the soil food web and are a suitable indicator for assessing soil environments and ecosystems. Previous nematode community analyses based on nematode morphology classification have been shown to be useful for assessing various soil environments. Here we have conducted DNA barcode analysis for soil nematode community analyses in Japanese soils. We isolated nematodes from two different environmental soils of an unmanaged flowerbed and an agricultural field using the improved flotation-sieving method. Small subunit (SSU) rDNA fragments were directly amplified from each of 68 (flowerbed samples) and 48 (field samples) isolated nematodes to determine the nucleotide sequence. Sixteen and thirteen operational taxonomic units (OTUs) were obtained by multiple sequence alignment from the flowerbed and agricultural field nematodes, respectively. All 29 SSU rDNA-derived OTUs (rOTUs) were further mapped onto a phylogenetic tree with 107 known nematode species. Interestingly, the two nematode communities examined were clearly distinct from each other in terms of trophic groups: Animal predators and plant feeders were markedly abundant in the flowerbed soils, in contrast, bacterial feeders were dominantly observed in the agricultural field soils. The data from the flowerbed nematodes suggests a possible food web among two different trophic nematode groups and plants (weeds) in the closed soil environment. Finally, DNA sequences derived from the mitochondrial cytochrome oxidase *c* subunit 1 (COI) gene were determined as a DNA barcode from 43 agricultural field soil nematodes. These nematodes were assigned to 13 rDNA-derived OTUs, but in the COI gene analysis were assigned to 23 COI gene-derived OTUs (cOTUs), indicating that COI gene-based barcoding may provide higher taxonomic resolution than conventional SSU rDNA-barcoding in soil nematode community analysis.

## Introduction

Nematodes are one of the most abundant metazoans on the Earth and are universally found in freshwater, terrestrial and marine environments [1] and even in the deep sea [2]. They exhibit various feeding types and survival strategies, for example, free-living bacterial and fungal feeders, predators, or animal and plant parasites. Nematodes are involved in the recycling of organic materials in soil and affect plant growth [3], [4]. Therefore, nematode feeding activity contributes to maintaining the integrity of soil food web. It is also well known that nematode plant parasites inhibit plant growth and crop production in farmland soils and can cause serious damage in agriculture [5]. Nematodes are suitable indicators for monitoring soil environments and the dynamics of nematode populations reflects nutrient conditions or toxicity in the soils (e.g., reviewed in [6]–[8]). For instance, Urzelai et al. examined the nematode communities in contaminated soils and suggested a possible good indicator of plant parasite index for monitoring recovery processes after perturbation [9]. Heininger and the colleagues found that nematode communities in the sediments were affected by the levels of pollution and showed that the genera composition of nematodes is useful as an indicator for assessing sediment pollution [10]. Nematode communities in various soils have been extensively studied in agricultural and environmental sciences, mainly by the morphology and/or feeding habit-based classification. These analyses require excellent taxonomic skills as well as a great deal of knowledge and experience, and exhibit serious limitations in terms of taxonomic identification and low sample throughput.

To overcome these bottlenecks in traditional morphology-based analyses, several PCR- or sequence-dependent molecular biological methods (e.g., denaturing gradient gel electrophoresis (DGGE) [11]–[13] and DNA barcoding) and spectroscopic techniques [14] have been developed for nematode taxonomic studies (also reviewed in [15]). Especially, DNA barcoding is based on interspecific differences of nucleotide sequences from a particular DNA region (i.e., DNA barcode sequences) and has been used as a powerful tool for taxonomic identification of eukaryotes and/or for their community analysis in various ecosystems [16]–[18]. In these studies, DNA sequences from the small subunit (SSU) or large subunit (LSU) rDNAs, mitochondrial cytochrome *c* oxidase 1 (COI) gene, and the internal transcribed sequence (ITS) of rDNA have been preferentially used as DNA barcodes. DNA barcoding has been an effective tool for taxonomic and community studies of soil animals including nematodes [19], [20]. The operational taxonomic units (OTUs) are generated by multiple-alignment of nematode DNA barcode sequences and nematodes aligned to the same OTU are assumed to be in the same taxonomic group. In addition, numbers of OTUs and abundance of nematodes in each OTU provide us with important qualitative and quantitative information on the nematode community: The former represents the number of taxonomic groups (i.e., variation of the species) and the latter shows the proportion of nematodes in each OTU within the entire nematode population present. In addition to taxonomic studies, a DNA barcode technique has been applied to analyzing the community structures of terrestrial and marine nematodes in various environments [21]–[28].

In addition to the assessment of soil environments [9], [10], soil nematode community analyses have previously showed their utility in biological assessments of environmental soils such as agricultural lands [7], [29]–[36]. However, these previous studies have been dependent on traditional morphology-based classification of nematodes by microscopic observations. So far, only a few studies using DNA barcoding have been reported for analyzing the soil nematode community [22], [24], [25], [28].

Here, we have performed DNA barcoding community analysis of nematodes living in two different soil environments in Japan. By using the improved flotation-sieving method, nematodes were isolated efficiently from the soils. DNA fragments of SSU rDNA were amplified by PCR for direct sequencing, resulting in SSU rDNA-derived OTUs (designated as rOTUs). Taxonomic classification of nematodes in each rOTU was determined by assigning 29 rOTUs to the phylogenetic tree containing 107 reference nematode SSU rDNA sequences. The taxonomic analysis of the rOTUs revealed a strikingly contrasting nematode population in the two soil environments: plant feeding and animal predatory nematodes were most abundant in the unmanaged flowerbed soil, whereas bacterial feeding nematodes were most abundant in the soybean-cultivated field soil. Finally, community analysis of the agricultural field soil nematodes was performed using two DNA barcode genes (SSU rDNA and the COI gene). The COI barcode analysis provided a more detailed nematode community structure rather than the conventional SSU rDNA-based analysis.

## Materials and Methods

### Study sites and soil sampling

Soil samples were collected between November 2010-January 2011 (under comparable climatic conditions) from a flowerbed framed in by concrete blocks and an agricultural field cultivated with soybean at the campus of Toyohashi University of Technology (Toyohashi Tech.), Toyohashi, Japan (137°24′E, 34°42′N [longitude: 137.4086, latitude: 34.7017]). The area of the flowerbed and the agricultural field were 1.19 m^2^ (0.7×1.7 m) and 2.38 m^2^ (0.7×3.4 m), respectively. The distance between two sampling sites is approximately 400 m. The flowerbed was unmanaged and weed-strewn for several years with no addition of fertilizer or tillage regime. The experimental field was cultivated with soybean after tillage, soil neutralization, and application of chemically synthesized fertilizer. Soil on the site was sampled to a depth of 15–20 cm using a 2.5 cm-diameter soil sampling auger (Fujiwara Scientific Co., Tokyo, Japan) and more than 3 independent soil samples were taken and mixed from each site. Average pH and water content of soils were 6.9±0.2 and 26.8±1.9% (flowerbed soil) and 8.1±0.1 and 22.8±0.6% (agricultural field soil), respectively. pH was measured using soil suspension with distilled water (soil∶water = 1∶2.5) and water content was determined by measuring the weight of dried soil (after drying at 65°C overnight). After removal of over-sized contaminants (e.g., stones) by passing soil samples through a 0.7 mm sieve, ca. 10 g of fresh weight soil was directly used for nematode isolation.

### Nematode isolation and DNA preparation

In the early stages of this study, nematodes were isolated from soil samples by a standard Baermann funnel method [37], however, the nematode isolation procedure was revised to increase the nematode isolation efficiency. An improved flotation sieving method using colloidal silica [38] was used in this study with some modifications. In brief, 10 g soil was made up to 40 ml with distilled water in a conical tube and gently mixed. After centrifugation at 1800× *g* for 5 min, the supernatant was poured off and 20 ml of 50% colloidal silica (LUDOX TM-50, Sigma-Aldrich, St. Louis, MO, USA) was added to the remaining soil pellet, followed by centrifugation at 1800× *g* for 15 min. The resulting supernatant was passed through a 160 µm nylon sieve (47 mm diameter, Millipore, Billerica, MA, USA) equipped with a filter holder (type KP-47S, ADVANTEC, Tokyo, Japan) and the flow-through fraction was then passed through a second, 20 µm nylon sieve (Millipore). Nematodes trapped on both sieves were eluted into water in a watch glass. Individual soil nematodes were randomly picked up and transferred into DNA LoBind tubes (Eppendorf, Hamburg, Germany) under a SZX16 stereomicroscope (Olympus, Tokyo, Japan). Single nematodes in individual tubes were subjected to subsequent DNA preparation [24]. Individual nematode in a tube was heated at 99°C for 3 min in 20 µl of 0.25 M NaOH and then neutralized by adding 4 µl of 1 M HCl, 10 µl of 0.5 M Tris-HCl (pH 8.0), and 5 µl of 2% Triton X-100. DNA samples were stored at −20°C until use. Each nematode isolated from flowerbed and agricultural field soils was designated as SnTUT_k01 and SnTUT_h01 with a two-digit serial number (e.g., SnTUT_k01_01), respectively.

### PCR amplifications

The 18S small subunit ribosomal RNA (SSU) gene fragments (approximately 900 bp) were amplified in 20 µl-reactions containing 10 µl of 2× PCR buffer for KOD FX Neo, 4 µl of 2 mM dNTPs, 0.4 unit of KOD FX Neo DNA polymerase (Toyobo, Tokyo, Japan), 2 µl of DNA sample, using 0.3 µM each of primers SSU18A-4F (5′-GCTTGTCTCAAAGATTAAGCCATGCATG-3′) and SSU26Rplus4 (5′- AAGACATTCTTGGCAAATGCTTTCG-3′). Amplification was initiated with a 2-min denaturation at 94°C followed by 35 cycles each involving denaturation at 94°C for 10 s, annealing at 55°C for 30 s, and extension at 68°C for 1 min. Prior to the experiments, several sets of primers were tested to determine if PCR products were reliably amplified from the nematode DNAs. A pair of PCR primers (SSU18A-4F and SSU26Rplus4) for the 900 bp-region of SSU rDNA (corresponding to the positions 963–1865 in the sequence of *C. elegans* rDNA cluster [accession no. X03680]) were selected and generated from the primers reported by Blaxter et al. [21] with modifications. The DNA fragments (approximately 400 bp) of mitochondrial COI gene were generated using primers COI_jb3-F (5′- GATTTTTTGGTCAYCCGGARGT-3′) and COI_jb5-R (5′- GYAACTACATAATAAGTRTCRTG-3′). Amplification for COI was initiated with a 2 min denaturation at 94°C, then a touch-down for 5 cycles with each cycle consisting of denaturation at 98°C for 10 s, annealing at 50, 49, 48, 47, and 46°C for 30 s, and extension at 68°C for 30 s, followed by 35 cycles of 98°C for 10 s, annealing at 45°C for 30 s, and extension at 68°C for 30 s. We initially tested the standard PCR primer set of LCO1490 and HCO2198 [17], however, PCR amplifications of the COI gene fragment from several soil nematode DNAs failed using these primers. Therefore, we generated new primers from several nematode COI gene sequences in the database and found that our newly designed primer set (as described above), derived from the I3-M11 partition in marine nematodes [23], can amplify COI fragments more efficiently than other primers. The forward and reverse primer sequences correspond to the positions 8572–8593 and 8967–8989 of the *C. elegans* mitochondrion genome nucleotide sequence (accession no. NC_001328). PCR amplifications were visually assessed by 1% agarose gel electrophoresis using 5 µl of each reaction.

### DNA sequencing

PCR products were purified from the reaction mixture using Illustra GFX PCR DNA and gel band purification kits (GE Healthcare UK Ltd., Buckinghamshire, England) following the manufacturer's protocol. When nonspecific amplification products were detected in PCR reactions, the specific products were purified from agarose gels with the kit. DNA sequences of both strands of purified PCR fragment were determined by direct sequencing. Sequencing reactions in both directions were performed using the BigDye Terminator v3.1 cycle sequencing kit with the same primers as used for PCR. Sequencing products were subsequently purified using BigDye XTerminator purification kit and analyzed in an ABI3130 genetic analyzer (Applied Biosystems, Foster City, CA, USA) following the manufacturer's instructions. Two inner primers for SSU rDNA (SSUF22: 5′-TCCAAGGAAGGCAGCAGGC-3′, SSUR09: 5′-AGCTGGAATTACCGCGGCTG-3′) were used for re-sequencing PCR products where the chromatogram contained ambiguous sequence. Chromatograms were trimmed and assembled using the ATGC software (version 4 for Macintosh, Genetyx Co., Tokyo, Japan) to obtain consensus sequences. For assembling sequences, unidirectional sequences were only used when high quality chromatograms with no double peak patterns and high fluorescence signals were obtained. Double peaks in both strands derived from multiple SSU rDNA alleles were confirmed by re-sequencing of the PCR product. DNA sequences (approximately 700–760 bp of SSU rDNA and 370 bp of COI) were deposited at DDBJ under the following accession numbers, AB728324-AB728482.

### Identification of operational taxonomic units (OTUs)

DNA sequence homologies among the nematode sequences were systematically examined using the GENETYX-MAC (version 16) and ATSQ software (Genetyx Co., Tokyo, Japan) to identify the operational taxonomic units (OTUs) containing almost identical sequences of the SSU rDNA and COI genes. Sequences with more than 99.5% identify were defined to be in the same OTU. The OTUs derived from SSU rDNA and COI gene sequences were designated as rOTU and cOTU, respectively. Each rOTU obtained from the flowerbed and agricultural field soil nematodes was named as K01rOTU and H01rOTU plus a serial two-digit number, respectively. The cOTUs were further refined by aligning the COI gene sequences of 43 nematodes isolated from agricultural field soil whose rOTUs were clarified.

### Phylogenetic analysis

FASTA format files containing SSU rDNA sequences of 16 K01rOTUs, 13 H01rOTUs, 107 publicly available reference nematodes and 4 outgroup species (Table S1) were prepared and these sequences were aligned using the ClustalW2 algorithm [39] in the software SeaView (version 4.2.12 for Macintosh) [40]. After sequence trimming, alignment of these sequences was subsequently performed using ClustalW2. A phylogenetic tree was constructed from the alignment file of SSU rDNA sequences using the neighbor-joining algorithm with Kimura two-parameter distance (bootstrap: 1000 replicates) in the SeaView program. The resultant tree file was used for drawing a cladogram using the GENETYX-Tree software (Genetyx Co., Tokyo, Japan). 107 reference nematodes were selected from previous taxonomic studies of terrestrial and marine nematodes to cover the orders in phylum Nematoda [21], [24], [26]. The outgroup consisted of *Dilta littoralis* (Arthropoda), *Gordius aquaticus* (Nematomorpha), *Priapulus caudatus* (Priapulida) and *Thulinia stephaniae* (Tardigrada). Phylogenetic analyses of K01rOTUs and H01rOTUs were also performed to draw unrooted trees as described above.

## Results

### Isolation of nematodes from the flowerbed and agricultural field soils

**Figure 1:**
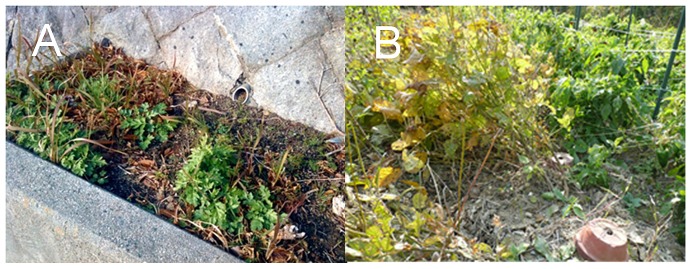
Photograph of the flowerbed and agricultural field sites for soil sampling. The unmanaged flowerbed framed by concrete walls (A) and the soybean-cultivated field (B) in the campus of Toyohashi University of Technology from autumn till winter in 2010.

The soils for nematode isolation were sampled from two different sites on the campus: an isolated and unmanaged flowerbed surrounded by concrete blocks (Fig. 1A), and an agricultural field cultivated with soybean (Fig. 1B) (see Materials and Methods for details about two sampling sites). Nematodes used for these experiments were isolated from the soils at the depth of 15–20 cm by the improved method based on centrifugal flotation and sieving in place of a standard Baermann funnel method [37]. Prior to the study, we examined numbers and morphological variation of nematodes isolated by these two methods, and found that the former improved method allowed us to isolate an increased number of various kinds of nematodes compared with the Baermann funnel method. For example, although 10±8.2 nematodes were isolated from 10 g of flowerbed soil by the conventional funnel method, higher numbers of nematodes (40.7±4.2) were consistently recovered from the same soil by the improved flotation and sieving method. Nematode density in the soybean-cultivated field soil was 3 to 4 fold higher than that in the flowerbed soil (data not shown).

### SSU rDNA barcode sequencing of soil nematodes and phylogenetic analysis

DNA samples were prepared by lysing nematodes with alkaline and heat treatments. 80 and 56 DNA samples were prepared from the nematodes isolated from the flowerbed and agricultural field soils, respectively. 68 and 48 nematode DNA sequences of 900 bp-SSU rDNA fragments from the flowerbed and agricultural field soils were successfully determined. Finally, the operational taxonomic units (OTUs) were generated by multiple sequence alignments with these SSU rDNA sequences, and 16 and 13 rOTUs (named as OTUs derived from SSU rDNA sequences) were obtained from flowerbed and agricultural field nematodes, respectively (Table 1). Each rOTU generated from the flowerbed and agricultural field nematodes was named as K01rOTU and H01rOTU with a two-digit number such as K01rOTU01 or H01rOTU01, respectively. The sequence identity between K01rOTU02a and K01rOTU02b was very close to the border value of 99.5% (i.e., 99.47%, 4 bp difference in 763 bp), and both rOTUs were distinguished by the names of K01rOTU with the same two-digit number (02) with a distinct letter. Several allelic sites were detected in the sequences from the flowerbed and agricultural field nematodes (Tables S2 and S3). These sites were reproducibly found as double peaks in the PCR product sequence chromatograms and were determined to be derived from different alleles.

**Table 1:** Summary of SSU rDNA barcode analysis of nematodes isolated from flowerbed and agricultural field soils.

rOTU^a^	No. of isolates	Nematodes belonging to each rOTU^b^	Feeding types^c^	Accession no.
K01rOTU01	18	SnTUT_k01_01, 05, 06, 08, 11, 12, 16, 18, 19, 24, 26, 29, 44, 52, 54, 65, 69, 75	plant feeding (1d)	AB728372–AB728389
K01rOTU02a	18	SnTUT_k01_03, 07, 14, 21, 32, 41, 42, 45, 51, 56, 63, 64, 66, 67, 68, 74, 76, 80	animal predation (5a)	AB728390–AB728407
K01rOTU02b	6	SnTUT_k01_22, 43, 55, 58, 59, 62	animal predation (5a)	AB728408–AB728413
K01rOTU03	6	SnTUT_k01_23, 33, 46, 49, 60, 61	animal predation (5)	AB728414–AB728419
K01rOTU04	4	SnTUT_k01_28, 40, 53, 73	plant feeding (1e)	AB728420–AB728423
K01rOTU05	3	SnTUT_k01_02, 20, 48	bacterial feeding (3)	AB728424–AB728426
K01rOTU06	3	SnTUT_k01_25, 36, 71	animal predation (5)	AB728427–AB728429
K01rOTU07	2	SnTUT_k01_70, 77	plant feeding (1d)	AB728430, AB728431
K01rOTU08	1	SnTUT_k01_34	plant feeding (1d), animal predation (5), omnivorous (8)	AB728432
K01rOTU09	1	SnTUT_k01_35	bacterial feeding (3)	AB728433
K01rOTU10	1	SnTUT_k01_37	plant feeding (1), hyphal feeding (2)?	AB728434
K01rOTU11	1	SnTUT_k01_39	animal predation (5), omnivorous (8)	AB728435
K01rOTU12	1	SnTUT_k01_47	-	AB728436
K01rOTU13	1	SnTUT_k01_50	-	AB728437
K01rOTU14	1	SnTUT_k01_57	-	AB728438
K01rOTU15	1	SnTUT_k01_72	-	AB728439
H01rOTU01	8	SnTUT_h01_02, 15, 21, 29, 38, 41, 43, 46	bacterial feeding (3)	AB728324–AB728331
H01rOTU02	6	SnTUT_h01_05, 19, 24, 26, 27, 40	bacterial feeding^d^	AB728332–AB728337
H01rOTU03	6	SnTUT_h01_22, 25, 30, 48, 54, 55	bacterial feeding (3)	AB728338–AB728343
H01rOTU04	5	SnTUT_h01_08, 23, 47, 50, 52	bacterial feeding (3)	AB728344–AB728348
H01rOTU05	5	SnTUT_h01_03, 14, 16, 33, 37	bacterial feeding (3)	AB728349–AB728353
H01rOTU06	5	SnTUT_h01_10, 13, 42, 51, 53	bacterial feeding (3)	AB728354–AB728358
H01rOTU07	3	SnTUT_h01_20, 44, 45	bacterial feeding (3)	AB728359–AB728361
H01rOTU08	2	SnTUT_h01_09, 35	bacterial feeding (3)	AB728362, AB728363
H01rOTU09	2	SnTUT_h01_04, 18	animal predation (5a)	AB728364, AB728365
H01rOTU10	2	SnTUT_h01_31, 32	-	AB728366, AB728367
H01rOTU11	2	SnTUT_h01_36, 39	hyphal feeding (2) or plant feeding (1e)	AB728368, AB728369
H01rOTU12	1	SnTUT_h01_01	-	AB728370
H01rOTU13	1	SnTUT_h01_17	animal predation (5)	AB728371

a Codes “K01”, “H01” and “rOTU” represent “flowerbed sample with experimental code” , “agricultural field sample with experimental code” and “SSU rDNA-derived OTU”, respectively. Two-digit serial numbers were assigned in order of the number of nematodes in the rOTU.

b Nematodes isolated from flowerbed and agricultural field soils were designated as SnTUT_k01_a two-digit serial number and SnTUT_h01_a two-digit serial number, respectively.

c Feeding types were derived from those of nematode species with the highest sequence homologies by Blast search according to the references in Yeates et al. (1993) [41]. Numbers and letters in parentheses indicate feeding types in the reference.

d Feeding type for H01rOTU02 was derived from the other reference [44].

**Figure 2:**
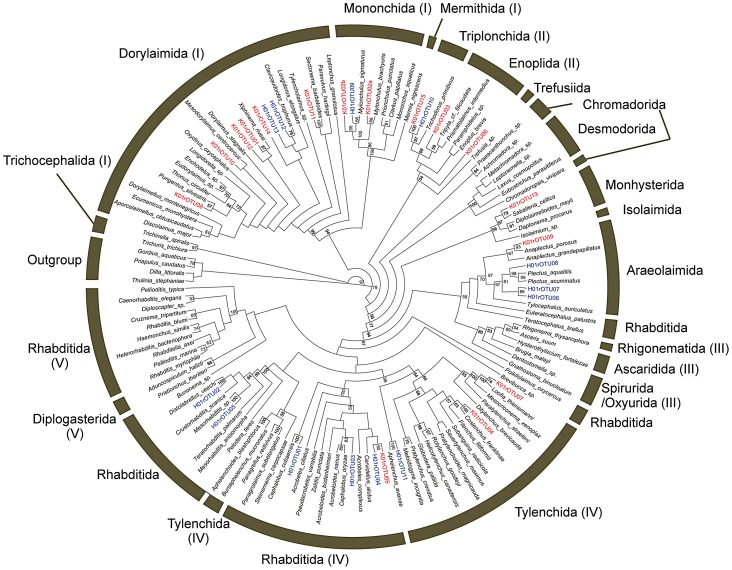
Neighbor-joining tree of SSU rDNA barcode sequences of soil nematodes and reference nematode species. SSU rDNA barcode sequences of 29 rOTUs from flowerbed and agricultural field soil nematodes (designated with K01rOTU and H01rOTU, and shown in red and blue, respectively) were analyzed with the corresponding SSU rDNA sequences of 107 reference nematodes (Table S1) and the resultant tree is displayed as a cladogram. Orders corresponding to the reference nematode species are indicated on the outside of the cladogram. The clade numbers (I–V) in the previous phylogenetic tree [21] were also indicated in parenthesis. Numbers on nodes are bootstrap values (>50%). *Dilta littoralis* (Arthropoda), *Gordius aquaticus* (Nematomorpha), *Priapulus caudatus* (Priapulida), *Thulinia stephaniae* (Tardigrada) were used as outgroup species.

The 29 rOTUs obtained from the nematodes in two different soils were further assigned into their taxonomic positions in phylum Nematoda by a phylogenetic analysis. The cladogram of the resulting SSU rDNA-based phylogenetic tree was shown in Figure 2. Twelve out of sixteen K01rOTUs from the flowerbed nematodes were mapped to the orders of Dorylamida (6 rOTUs), Mononchida (2 rOTUs), Enoplida (2 rOTUs), and Tylenchida (2 rOTUs). In contrast, 10 out of 13 H01rOTUs from the agricultural soil nematodes were assigned to the orders of Rhabditida (5 rOTUs), Araeolaimida (3 rOTUs), and Dorylamida (2 rOTUs). These clearly indicate distinct nematode communities in the two soils.

### Two soil nematode communities with different trophic types

**Figure 3:**
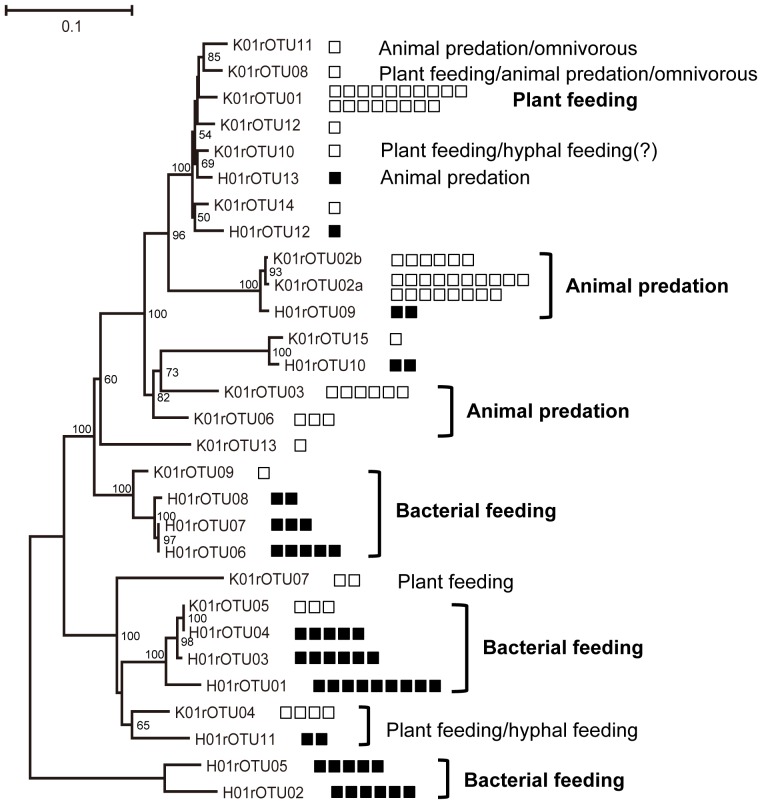
An unrooted phylogram of rOTUs from soil nematodes isolated from the flowerbed and agricultural field with predicted trophic types. Twenty-nine SSU rDNA barcode sequences of K01rOTUs (flowerbed samples) and H01rOTUs (agricultural field samples) were aligned for preparing a phylogenetic tree. The numbers of soil nematodes belonging to each rOTU correspond to the numbers of open (flowerbed samples) and closed (agricultural field samples) squares at the right. Numbers on nodes are bootstrap values (>50%). Trophic types indicated in the rOTUs were derived from those of the nematode species with the highest homology in SSU rDNA barcode sequences (Table 1), and the feeding types for the rOTUs containing a large number of nematode members are shown in boldface. Bar: 0.1 substitutions per site.

To further investigate the taxonomic differences between the two soil nematode populations, the 29 rOTUs from both samples were classified by their sequence homologies and the resulting unrooted phylogenetic tree of the rOTUs is shown together with predicted trophic types in Figure 3. One pair of rOTUs (i.e., K01rOTU05 and H01rOTU04) was common between the two nematode communities, whereas all other rOTUs identified were not common between the two soils. According to a previous study on nematode feeding behaviors [41], we determined feeding habits (trophic types) for each rOTU as those of the nematode species with the highest homologies to the rOTU sequence by the BLAST search (Tables S2 and S3). Many nematodes from the flowerbed soil were assigned to animal predation and plant feeding trophic types. On the contrary, nematodes from the agricultural field soil (closed squares) were mostly related to bacterial feeding nematodes (bacteriovores) (Fig. 3). For example, the SSU rDNA barcode sequence of K01rOTU01, containing a large number (18) of members, completely matches those of the representative plant-parasitic nematode *Xiphinema* species [42] by homology search (100% sequence identity in the entire 766 bp-region). Two related rOTUs of K01rOTU2a and K01rOTU2b containing a total of 24 isolates shared the highest sequence homologies with the SSU rDNA sequences of the predatory nematode species of the genus Mylonchulus (Table S2) [43]. About 60% of isolates (42 out of 68) from the flowerbed soil belong to these three K01rOTUs (01, 02a and 02b). Based on the SSU rDNA sequence homology search, 6 and 4 nematodes in the next largest K01rOTUs (K01rOTU03 and K01rOTU04) were also assigned to the genera Tripylella and Coslenchus, with predacious and plant feeding behaviors [41], respectively.

In the soybean-cultivated field soil, the six H01rOTUs containing more than 5 isolates were assigned to genera in the families Cephalobidae (H01rOTU01, H01rOTU03, and H01rOTU04), Rhabditidae (H01rOTU02 and H01rOTU05), and Plectidae (H01rOTU06). According to a previous study [41], [44], these six H01rOTUs, representing approximately 60% of total sequenced nematodes from the agricultural field soil, were strongly suggested to have bacterial feeding habits (Table S3).

### SSU- and COI-barcode analyses of the nematode community in the agricultural field soil

In this study, we used a SSU rDNA sequence as a DNA barcode for analyzing the nematode community because SSU rDNA sequences have been widely used in previous studies and a large number of SSU rDNA sequences of nematode species are available in the public database. The mitochondrial COI gene sequence has also been a popular choice as a DNA barcode for phylogenetic analysis and community analysis in animals. Although there are limited numbers of nematode COI gene sequences deposited in the database, some studies have suggested that mitochondrial COI gene barcoding was useful for community analysis of nematodes [22], [23], [26]. Therefore, we performed a phylogenetic analysis of soil nematodes from the agricultural field soil using the COI gene barcode. By using the newly-designed primers (see Materials and Methods for details), PCR products of approximately 400 bp were obtained in the reactions in a relatively reproducible fashion. Then, we have determined the partial COI gene sequences from 48 nematodes (in Table S3) from the soybean-cultivated field in addition to their SSU rDNA barcode sequences. 43 out of 48 COI gene sequences (approximately 370 bp) were successfully recovered and aligned for further analysis. Twenty-three COI gene-derived OTUs (designated as H01cOTUs) were generated from the agricultural field nematodes (Table S4). By comparing with the 13 H01rOTUs generated in this study, almost twice as many H01cOTUs were generated from the same set of nematode DNAs, suggesting that the COI gene-based barcoding provides the nematode community analysis with higher resolution than the conventional SSU rDNA-based barcoding. Many of the H01cOTUs (16 of 23 H01cOTUs) only contained a single nematode as a member. The H01rOTUs and H01cOTUs obtained from 43 individual nematodes are summarized in Table 2. Eight out of 13 H01rOTUs include multiple H01cOTUs. Most of the H01cOTUs correspond to distinct H01rOTUs, however, 5 nematodes assigned to H01cOTU02 were separately classified to 3 different H01rOTUs: one isolate each for H01rOTU03 and H01rOTU04, and 3 isolates to H01rOTU05, respectively. This complicated pattern in SSU rDNA- and COI gene-based OTUs has been also observed in the previous taxonomic study of tardigrade specimens by Blaxter et al. [22], and may correspond to particularly variable single, or diverging taxonomic groups. This pattern may reflect divergence of matrilineal lines due to asexual reproduction in nematodes as described previously [22].

**Table 2:** Summary of SSU rDNA- and COI gene-barcode analyses using nematodes from the agricultural field soil.

H01rOTU^a^	Name of nematode	H01cOTU	No. of nematodes in each H01rOTU	Corresponding H01cOTUs^b^
01	SnTUT_h01_02	01	8	01, 19, 21
	SnTUT_h01_15	01		
	SnTUT_h01_21	01		
	SnTUT_h01_29	01		
	SnTUT_h01_38	19		
	SnTUT_h01_41	21		
	SnTUT_h01_43	01		
	SnTUT_h01_46	01		
02	SnTUT_h01_05	03	5	03, 15
	SnTUT_h01_19	15		
	SnTUT_h01_24	03		
	SnTUT_h01_26	03		
	SnTUT_h01_27	03		
03	SnTUT_h01_22	05	5	*02* c, 05
	SnTUT_h01_25	05		
	SnTUT_h01_30	05		
	SnTUT_h01_48	02		
	SnTUT_h01_54	05		
04	SnTUT_h01_08	10	5	*02*, 06, 10, 16, 23
	SnTUT_h01_23	16		
	SnTUT_h01_47	02		
	SnTUT_h01_50	23		
	SnTUT_h01_52	06		
05	SnTUT_h01_03	02	4	*02*, 12
	SnTUT_h01_16	12		
	SnTUT_h01_33	02		
	SnTUT_h01_37	02		
06	SnTUT_h01_10	11	5	04, 11
	SnTUT_h01_13	04		
	SnTUT_h01_42	04		
	SnTUT_h01_51	04		
	SnTUT_h01_53	04		
07	SnTUT_h01_20	08	3	08, 22
	SnTUT_h01_44	22		
	SnTUT_h01_45	08		
08	SnTUT_h01_09	07	2	07
	SnTUT_h01_35	07		
09	SnTUT_h01_18	14	1	14
10	SnTUT_h01_32	17	1	17
11	SnTUT_h01_36	18	2	18, 20
	SnTUT_h01_39	20		
12	SnTUT_h01_01	09	1	09
13	SnTUT_h01_17	13	1	13

a Two-digit number of each H01rOTU is indicated. The description of two-digit number was omitted in the following rows containing nematodes belonging to the same H01rOTU.

b Nematodes in H01rOTU in the left-most column belong to the H01cOTUs with two-digit numbers shown.

c Nematodes in H01cOTU02 were separately assigned to H01rOTU03-H01rOTU05 and are indicated in italics.

## Discussion

DNA barcode analysis is a powerful tool for clarifying the composition and taxonomic identity of organisms in the environment. Previous studies of DNA barcoding have been mainly focused on taxonomic identification and classification of organisms. In this study, we have performed DNA barcode-based analyses of the nematode community living in two ordinary Japanese soil environments: an unmanaged and isolated flowerbed and a typical agricultural field cultivated with soybean.

Since efficient isolation of the nematodes living in soils is very important for further community analysis, prior to the study, we referred to previous studies on improved isolation of soil nematodes with colloidal silica extraction and/or sieving [38], [45]–[49], and further modified a flotation and sieving method in place of the commonly used Baermann funnel method [37]. By using this method, higher numbers (ca. 3–4 fold) of total nematodes were obtained when compared with the Baermann funnel method. In addition, nematodes closely related to the family Hemicycliophoridae with relatively poor locomotion were successfully isolated only by the improved method (e.g., two nematodes in K01rOTU07) (Table S2). Due to improved recovery of nematodes, the nematode population isolated in this study is considered to be suitable for the community analysis.

Nematode community analyses are useful for assessing soil environments and several extensive biological assessments have been conducted for various soil environments in Europe, the United States, Asia, Australia and New Zealand [7], [29]–[36]. These analyses were performed based on the traditional morphological classification of nematodes under the microscope. In spite of recent progress in DNA barcoding, a limited number of studies on terrestrial nematode community analyses have been reported. To date, SSU rDNA barcoding to assess the community structure of soil nematodes have been undertaken from a barley field soil [25], a hill farm grassland soil [24], and moss ecosystems in the UK [22]. In addition, Powers and colleagues applied the SSU-based DNA barcoding to analyze nematode communities in a tropical rainforest in Costa Rica and found potentially novel taxonomic groups [28]. Although DNA barcode-mediated taxonomic identifications of soil nematode species have been reported in Japan [50]–[52], nematode community analyses in Japanese soils with DNA barcoding have not been undertaken. In this study, we first analyzed the nematode communities in two different types of soils in Japan by DNA barcoding, and found contrasting community structures in these soils. The flowerbed soil was dominated by animal predators and plant feeders that occupied more than 80% of total nematodes (Fig. 3 and Table 1), strongly suggesting a possible food web including two nematode populations and plants (weeds) in the unmanaged and isolated flowerbed soil environment. Plant feeding nematodes propagate by living with weeds in the flowerbed and predators likely prey on these plant-parasitic nematodes in a possible food chain.

In the soybean-cultivated field soil, bacterivores were the dominant nematode type isolated (Fig. 3 and Table 1). Previous studies have examined nematode communities in agricultural soils. Okada and Harada analyzed the taxonomy of nematodes in a Japanese soybean field with different treatments by morphological classification in order to examine the effects of tillage and fertilizer [32]. In this study, two abundant nematode genera Acrobeloides (bacterial feeder) and Pratylenchus (root feeder) were observed as well as three other predominant bacterial feeders of the families Rhabditidae, Plectidae, and Prismatolaimidae. Baird and Bernard also examined the population and dynamics of nematodes in soybean-wheat cropping regimes and reported that approximately 80% of total nematodes were almost evenly classified to three groups, plant-parasites, Dorylaimida (the order including plant-parasites, omnivores and fungivores) and bacterivores [53]. Neher et al. characterized the soil nematode communities in three different ecosystems (agricultural land, forest and wetland) [54]. They also showed abundant bacterial feeding nematodes in the families Cephalobidae and Rhabditidae in agricultural soils, similar to those observed in the soybean-cultivated field soil in our study. From these previous studies and our own current study, we have shown that abundant bacterial feeding nematodes are characteristically observed in agricultural soils.

Interestingly, Neher et al. found nematodes from the families Criconematidae, Hoplolaimidae, and Oxydiridae (plant-parasites) and Nygolaimidae (predators) as major populations in undisturbed wetland soil [54]. Unlike in agricultural soils, plant feeder- and predator-dominant community structure observed in the wetland was commonly found in the unmanaged flowerbed in our study (Fig. 3). The distinct composition of nematodes in these ecosystems may reflect the difference in microbial biomass. Previous studies showed that a high abundance of bacterivores is associated with an increase in microbial organisms [33] and soil organic matter [55]. Yeates and King also examined the nematode populations in native and improved (fertilized) grasslands in Australia and found a significant increase in the proportion of bacterial feeding nematodes in the improved soils [34]. Although we did not measure microbial biomass and nutrients in our soils, a fertilized and soybean-cultivated field soil likely contains more abundant bacteria that are enough to support a large number of bacterivores rather than a weed-grown flowerbed soil without any supply of fertilizer. Unlike agricultural field soils with abundant biomass, bacterial feeding nematodes are unlikely to flourish in soils with limited biomass such as wetland and isolated flowerbed soils, however, plant-parasites that can live with some weeds and their predators could survive and grow in these soils.

In this study, we have compared the classification of a set of soil nematodes from the agricultural field by SSU rDNA- and COI gene-barcoding. As shown in Tables 2 and S4, COI gene sequences from 43 soil nematodes were classified to 23 cOTUs, a significant increase compared to the 13 rOTUs from SSU rDNA barcode sequencing. Nassonova et al. [56] recently reported that the COI-based barcoding of amoebae provided a higher taxonomic resolution than the SSU rDNA-based analysis, and it may be in agreement with our results. On the other hands, Blaxter et al. [22] compared the SSU rDNA- and COI-OTUs obtained from 82 tardigrades from moss ecosystems and showed that the larger number of SSU rDNA-OTUs (23) was detected than 17 of COI-OTUs. The disagreement among the studies may be derived from the difference of examined organisms or the different COI gene regions used. We also found some issues with using the COI gene-barcoding approach. For example, PCR with the conventional primer set (LCO1490 and HCO2198) from the M1-M6 region of COI gene [17] worked poorly with soil nematode DNAs under our experimental conditions and we had to develop novel primers (COI_jb3-F and COI_jb5-R) from another region of the COI gene (I3-M11 region) [23]. Derycke et al. also reported inefficient PCR with the conventional COI gene primers for barcode analysis of marine nematodes and developed new primers from the I3-M11 region [23]. Although we improved the primers and reaction conditions and succeeded in increasing the efficiency of PCR amplification in this study, this primer set is not still perfect for COI gene-barcoding studies. Amplification of the COI gene requires complicated PCR cycles and amplification is less efficient when compared with amplification of SSU rDNA fragments. Further improvements to the method may be required to optimize PCR for preparing COI gene fragments from various nematode species.

Recently, high-throughput next generation DNA sequencing has been used to accelerate DNA barcode-based community analyses for various eukaryotes to assess ecosystem biodiversity [57], [58]. This analysis primarily depends on cluster analysis with large numbers of sequences of PCR-amplified DNA fragments from organisms in the community and enables researchers to analyze a large number of organisms at once unlike a single isolate-based DNA barcode sequencing. Metagenomic analyses for nematode diversity were extensively performed using the next generation sequencer (the 454 GS FLX) by Porazinska et al. [59]–[61]. In these analyses, huge amounts of nucleotide sequence data were generated from PCR-amplified SSU rDNA fragments (ca. 400 bp in size). Unlike conventional DNA barcode analyses, thousands of OTUs have been obtained by clustering the sequences (more than 200 bp in each length). Although the nematode communities from multiple soil samples can be achieved in a high-throughput fashion, considerable issues still remain in these metagenomic analyses such as contamination by interspecific and intraspecific chimeric sequences and unclassified sequences and possible biased amplification of PCR products [60]. In place of traditional morphology-based methods, a conventional isolate-based DNA barcoding is sufficient to determine limited but accurate community structures of soil nematodes in ecosystems and agricultural soils for probing and assessing soil environments.

## Supporting Information

Table S1 **Nematode species used in the phylogenetic analysis and accession numbers.**
(XLS)

Table S2 **Summary of H01rOTUs of nematodes isolated from a flowerbed soil.**
(XLS)

Table S3 **Summary of H01rOTUs of nematodes isolated from an agricultural field soil.**
(XLS)

Table S4 **Summary of COI gene barcode analysis of nematodes isolated from an agricultural field soil.**
(XLS)
